# Stress-induced survival strategies enable *Salmonella *Enteritidis to persistently colonize the chicken oviduct tissue and cope with antimicrobial factors in egg white: A hypothesis to explain a pandemic

**DOI:** 10.1186/1757-4749-2-23

**Published:** 2010-12-20

**Authors:** Filip Van Immerseel

**Affiliations:** 1 Department of Pathology, Bacteriology and Avian Diseases, Research Group Veterinary Public Health and Zoonoses, Faculty of Veterinary Medicine, Ghent University, Salisburylaan 133, B-9820 Merelbeke, Belgium

## Abstract

**Background:**

Egg-associated transmission to humans seems to be characteristic of the *Salmonella *serotype Enteritidis, explaining why this particular serotype has caused a worldwide pandemic since the mid '80s. *Salmonella *Enteritidis is much more capable to persistently colonize the laying hen reproductive tract and to survive in the hostile egg white, as compared to other serotypes.

**Presentation of the hypothesis:**

It is hypothesized that stress-induced survival mechanisms enable the serotype Enteritidis to persistently colonize the oviduct without causing damage and excessive inflammation, and to cope with the antimicrobial compounds present in egg white.

**Testing the hypothesis:**

To test the hypothesis first of all *Salmonella *Enteritidis genes that are essential for colonization of the oviduct and survival in eggs need to be identified. Comparative genomics tools should be used to identify genes or pathogenicity islands that are present in *Salmonella *Enteritidis and not in the multiple non egg-contaminating serotypes. High-throughput signature-tagged-mutagenesis approaches, coupled to micro-array detection of the genes that lead to an attenuated phenotype when mutated is proposed as an ideal tool to identify genes involved in oviduct colonization and egg white survival. Identifying the stressors and antibacterial molecules in the oviduct and in the egg white that limit colonization or survival of non-Enteritidis serotypes is a second important objective that can theoretically be achieved using screenings of expressed oviduct cDNA libraries for their antibacterial activity against strains from multiple serotypes. Finally, the effect of contact with these stressors in the oviduct or egg white on *Salmonella *gene expression will need to be analyzed, in order to clarify whether serotype Enteritidis-specific regulation of certain stress-survival pathways are either or not present.

**Implications of the hypothesis:**

Knowledge on the pathogenesis of egg infections would furthermore give insights that might be extrapolated to other biological interactions, in which a highly specialized bacterial pathogen resists the host response in a specific biological niche. In addition, this info can be of value in developing early warning criteria to identify emerging egg-associated *Salmonella *strains and in developing safe live attenuated vaccine strains.

## Background

*Salmonella *is worldwide one of the most important causes of zoonotic disease, associated with consumption of contaminated food. Most important food vehicles for *Salmonella *are eggs and egg products. The serotype typically associated with eggs and egg products is *Salmonella *Enteritidis. This serotype is highly prevalent in live poultry, both in broilers and laying hens. In live chickens, also many other *Salmonella *serotypes are detected, but egg-associated transmission to humans seems to be a characteristic that is specifically reserved for the serotype *Salmonella *Enteritidis, explaining why this particular serotype has caused a worldwide pandemic since the mid '80s [1,2].

Eggs can be contaminated on the outer shell or internally. Outer shell contamination is the result of environmental contamination after shedding of the pathogen by the animal. Internal egg contamination can in principle occur following by eggshell and eggshell membrane penetration, but it is believed that most often colonization of the reproductive tract and incorporation in the forming egg is the main route [3]. Egg formation in the oviduct takes about 24 hours during which the sequential addition of molecules takes place in different compartments of the reproductive tract while the forming egg is migrating from the ovary to the vagina, before being laid. Indeed, the yolk is produced in the ovary, the infundibulum (most upper part of the oviduct) captures the yolk, the magnum segment produces the egg white, the isthmus deposits the eggshell membranes, the uterus forms the shell and the vagina is involved in oviposition. Depending on the oviduct segment in which *Salmonella *bacteria are present, the bacteria can thus be incorporated in different parts of the egg. Yolk contamination would lead to extensive growth [4]. This would result in a drop in egg production. Therefore it is generally believed that eggs are mostly contaminated in the egg white, in which antibacterial factors are present that limit bacterial multiplication.

Until now, it has not been clearly explained why the serotype Enteritidis specifically has been implicated in egg contamination. It is clear however that *Salmonella *Enteritidis can contaminate the reproductive tract and seems to be a better colonizer of the oviduct environment as compared to other serotypes [5]. Secondly, it has been shown by comparing different strains from multiple serotypes that in general, *Salmonella *Enteritidis strains cope better with the antimicrobial properties of egg white as compared to other serotypes [6]. More efficient oviduct colonization and better survival in egg white are ideal characteristics for a pathogen that is transmitted to egg-consuming hosts. Despite this knowledge, the molecular mechanisms behind this behavior of *Salmonella *Enteritidis are still not unraveled. Some *Salmonella *genes that are involved in oviduct colonization and egg white survival have been identified, but genome-wide screening tools, complemented with in-depth studies on the role of specific genes, would be most welcome to get a better profile of the interaction of *Salmonella *Enteritidis with the oviduct tissue and egg white. Genes shown to be highly expressed in the oviduct or essential for oviduct colonization that are described until now are mostly major virulence genes that contribute to colonization of any organ, and are not exclusively related to oviduct colonization [3]. In the egg white, numerous antimicrobial compounds have been identified. These include molecules that degrade microbial components, such as lysozyme, antibacterial peptides, such as avian β-defensins, lipopolysaccharide binding and bactericidal/permeability increasing proteins (LBP-BPI proteins) that bind LPS and permeabilize the cytoplasmic membrane, such as ovocalyxin-36, molecules decreasing bioavailability of cations and vitamins, such as ovotransferrin, and many others [7-9]. Strikingly, the activity of these molecules to *Salmonella *and the potential protection mechanisms of *Salmonella *against these molecules are very poorly investigated under the conditions encountered by the bacteria it the chicken reproductive tract.

In this paper, it is proposed that stress-induced survival mechanisms enable the serotype Enteritidis to persistently colonize the oviduct without inducing damage and excessive inflammation, and to cope with the antimicrobial compounds and thus survive in egg white. It is proposed that studies should be carried out to a) analyze the virulence mechanisms that allow *Salmonella *Enteritidis to persistently colonize the oviduct tissue and b) to identify egg white molecules that have antibacterial activity against *Salmonella *and unravel the mechanisms that protect *Salmonella *Enteritidis against these environmental insults, thus enabling its survival in egg white.

## Presentation of the hypothesis

The *Salmonella *serotype Enteritidis has caused a worldwide pandemic due to its ability to persistently colonize the oviduct tissue of laying hens and survive in the hostile egg white environment. The *Salmonella *Enteritidis O- and H-antigens, and thus the LPS and flagellar antigens, are discriminating Enteritidis from many other serotypes (from other serogroups). It could thus very well be that the LPS structure of *Salmonella *serogroup D, containing the serotype Enteritidis, could be more resistant to penetration of antibacterial molecules, such as cationic antimicrobial peptides (CAMPs) present in egg white. However, this does not explain why strains from serotype Enteritidis and not other serogroup D serotypes have a tropism for eggs. It is hypothesized that stress-induced survival mechanisms play a role in the ability to persistently colonize the oviduct and to cope with the antibacterial molecules present in egg white. In other words, it is proposed that the serotype Enteritidis either contains Enteritidis specific genes or a specific regulation of stress-induced genes that enable these strains to live in close association with the laying hen oviduct and the egg white. As an example, successful strains could colonize the oviduct without causing overt disease, inflammation and tissue damage, and thus without a drop in egg production, and be incorporated in eggs during passage in the oviduct. Once inside the egg, these strains could respond to the environmental stresses they encounter by hitherto undefined stress-induced protection mechanisms, such as cell wall modifications or mechanisms that are used to export antibacterial compounds.

## Testing the hypothesis

Testing of the hypothesis would require a multidisciplinary approach that, among others, should try to reply to the following questions: 1) which *Salmonella *Enteritidis genes are essential for colonization of the oviduct and survival in eggs?; 2) what are the stressors that *Salmonella *encounters in the oviduct and in the egg white and limit colonization or survival of other serotypes?; 3) what is the effect of contact with these stressors in the oviduct or egg white on *Salmonella *gene expression and does this transcriptome contain *Salmonella *Enteritidis specific gene responses or a *Salmonella *Enteritidis specific regulation of certain stress-survival pathways?

To identify *Salmonella *Enteritidis genes essential for oviduct colonization and egg white survival, different approaches, including many more than those proposed in this paper, can be used. First of all, one could analyze whether gene sequences that are present in Enteritidis but not in other serotypes exist and whether the encoded proteins have a role in the interactions with eggs or with the oviduct (cells). This would clearly require a comparative genomics approach using multiple serotype Enteritidis strains and strains from a variety of other serotypes. Earlier studies have already shown that no consistent large genomic differences exist between recent *Salmonella *Enteritidis isolates and isolated from the 1940's and 50's [10]. When comparing *Salmonella *Enteritidis with *Salmonella *Typhimurium and *Salmonella *Gallinarum, genes and genome islands were identified that were specific for the strains from the respective serotypes [11]. It has been shown that *Salmonella *Gallinarum is most likely a descendant of *Salmonella *Enteritidis, because both genomes are very similar with the exception that *Salmonella *Gallinarum has lost multiple genes and genomic islands during evolution [11]. This is of extreme interest because *Salmonella *Gallinarum is adapted to laying hens and, in contrast to Enteritidis, is not colonizing the gut to large extent, but colonizing and damaging the reproductive tract and other organs severely, resulting in clinical symptoms and death [12]. Searching for the function of the genes that are lost during *Salmonella *Gallinarum divergence would thus possibly give insights in the pathogenesis of egg infections, and result in the identification of genes that cause a less destructive phenotype, what would help in colonizing the reproductive tract without causing drops in egg production. Although the genomic comparisons with serotype Typhimurium strains can be interesting, this serotype has, although on a limited scale, also been implicated in egg infections and can clearly also colonize the oviduct of laying hens. It would however be of interest to compare the *Salmonella *Enteritidis genome with the genome of strains that are known not to persistently colonize the oviduct or contaminate eggs, such as Virchow, Hadar, other serogroup D strains, and multiple others. This may help to identify *Salmonella *Enteritidis specific genes that could potentially be involved in the adaptation to the oviduct and egg environment. Another way to identify genes involved in oviduct colonization or egg white survival could be the use of signature-tagged mutagenesis (STM)-related approaches, such as the microarray-based negative selection strategy that has been used by different authors to identify genes involved in colonization of mouse organs [13,14]. This method is based on the inoculation of a transposon-mutagenized library of *Salmonella *in a live animal, and a micro-array based detection of all transposon-inserted sequences in the *Salmonella *isolates recovered from the organ. Selective disappearance of mutants will thus yield a list of genes that are essential to colonize an organ. This method could clearly be of use to identify *Salmonella *Enteritidis genes involved in chicken oviduct colonization. Whatever method is used, it is clear that analyzing the behavior of deletion mutants in the genes that are identified by genomic comparisons or STM-related approaches as being potentially involved in oviduct colonization or egg white survival, will give the final proof for its actual role in these processes.

It is clear that the oviduct is a harmful environment for *Salmonella*. Indeed, *Salmonella *strains are colonizing any other organ, including the gut, liver and spleen, much better than the oviduct [5,15,16]. Moreover, metabolic mutants can colonize the gut and internal organs much longer than they can colonize the oviduct [17]. Egg white, or oviduct mucus, is clearly antibacterial and many antibacterial molecules from egg white have been described, but the anti-*Salmonella *activity of these molecules is under-investigated. It has been shown that *Salmonella *Enteritidis if far more capable to survive in egg white compared to strains from other serotypes [6], but the actual reason is unclear. It does not seem to relate only to the structural differences in LPS structure, because strains from the same serogroup D also survive less. Knowledge on the mechanisms by which egg white reduces the *Salmonella *load in eggs and more important, data on the specific protection mechanisms of *Salmonella *Enteritidis against these egg white stressors, could enhance our understanding of the actual reasons for the egg-derived pandemic. The differential response of *Salmonella *serotypes and strains to certain egg white specific factors, such as alkaline pH (8 to 9), can easily be tested, but it is reasonable to believe that one or a subset of egg white components are involved in the bacteriostatic or bactericidal activity against *Salmonella*. Isolating all these components and testing them one by one would be an impossible task seen the complexity of egg white. Although a challenge, it would be an option to test all egg white proteins at once after cloning an inducible oviduct cDNA library in *Salmonella *and testing viability after induction of expression. Chicken reproductive tract cDNA libraries have already been used to identify antibacterial eggshell proteins [18,19]. Recently, a high-throughput screening method was developed to identify antibacterial peptides [20]. The method uses inducible library expression in *E. coli *combined with a dye inclusion assay to monitor bacterial cell viability. It is thus suitable to identify antibacterial peptides or proteins. If this system would be converted to *Salmonella *and if an oviduct cDNA library could be properly expressed in *Salmonella*, this could open perspectives for antibacterial egg white protein identification. Of specific interest would be proteins that are antibacterial for non-Enteritidis strains, but not for *Salmonella *Enteritidis. Important in this regard in the recent finding that a *Salmonella *Enteritidis mutant in tolC, the outer membrane channel linked to multi-drug resistance (MDR) efflux pumps, is required to survive in egg white [21]. It could well be that *Salmonella *Enteritidis is capable to export specific antibacterial egg white molecules, while this trait is not present in other serotypes.

Identifying *Salmonella *Enteritidis specific gene sequences or *Salmonella *Enteritidis specific genes involved in oviduct colonization or egg white survival, and identifying antibacterial molecules in egg white that affect non-Enteritidis serotypes, if existing, would be a difficult but feasible approach. Even more complex would be that the selective advantage of Enteritidis would be due to differences in expression levels of *Salmonella *genes, present in any serotype, but differentially regulated by environmental triggers in the different serotypes. Measuring the expression profile of the *Salmonella *genome in the oviduct or eggs is possible using micro-array systems. Systems such as the in vivo expression technology (IVET) have been used already to identify genes highly expressed in the chicken oviduct [17]. Although measuring expression in the oviduct or eggs is feasible, the challenge would be to evaluate the significance of this upregulation. If, in an ideal situation, anti-*Salmonella *molecules present in the oviduct or egg white would be identified that affect most, if not all, non-Enteritidis serotypes but not Enteritidis strains, then it would be fairly easy to study the transcriptome and analyze the response to these stressors.

## Implications of the hypothesis

It is astonishing that 30 years after the rise of the egg-associated pandemic caused by *Salmonella *Enteritidis, still the actual reasons for the close interaction of this serotype with eggs are not clarified. Methodologies now are in place to perform detailed studies on the molecular interactions between *Salmonella *strains, the oviduct tissue and egg white molecules. It is of utmost importance to understand the biological differences between the (un)ability of *Salmonella *strains and serotypes that are able vs those that are unable to colonize the laying hen oviduct and contaminate eggs. Published and unpublished data (from our own group and collaborators) point towards an interaction in which *Salmonella *Enteritidis is more capable to withstand the harmful environment of the oviduct and the attacks by antibacterial compounds present in egg white. It is hypothesized that this could be caused by stress-induced activation of a protection mechanism, what can be investigated using currently available methodologies. The implications of this hypothesis are multiple. First of all, identifying *Salmonella *Enteritidis specific mechanisms for persistent oviduct colonization and egg white survival would possibly aid in a more rapid identification of serotypes or strains that, in the future, would emerge as egg-associated strains. Thus a diagnostic tool to screen *Salmonella *strains for the ability to cause egg infections could be developed. Secondly, a potential application lies in genetic selection for laying hens that are capable to produce lethal concentrations of specific protective molecules in the egg white. It could well be that concentration dependant killing by an egg white molecule is responsible for killing of most *Salmonella *serotypes with the exception of *Salmonella *Enteritidis. In this case genetic selection could be used to select for a breed with higher concentrations of the antibacterial compound in the egg white. In addition, knowledge on genes essential to contaminate eggs is crucial for development of vaccines for laying hens. Genetically modified live attenuated vaccines could thus be developed without concerns that they would potentially contaminate eggs used for consumption. Knowledge on the pathogenesis of egg infections would furthermore give insights that might be extrapolated to other biological interactions, in which a highly specialized bacterial pathogen resists the host response in a specific biological niche.

## List of abbreviations

None.

## Competing interests

The authors declare that they have no competing interests.

## Authors' contributions

Prof. F. Van Immerseel is research professor (BOF-ZAP) and coordinating the activities of a research group working on the pathogenesis of egg infections by *Salmonella *and wrote the hypothesis paper.
